# Supplementary material for: Relying on the French territorial offer of thermal spa therapies to build a care pathway for long COVID-19 patients
Source: PLoS One. 2024 Apr 19;19(4):e0302392. doi: 10.1371/journal.pone.0302392 (PMC11029631; doi:10.1371/journal.pone.0302392)
Supplement: S1 Appendix — (DOCX) [file pone.0302392.s001.docx]

**S1 Appendix. Links to the public data used to produce figures 1 to 3**

Data used to produce figures 1 to 3 are public data and can be accessed via the following links:

- INSEE data can be used free of charge for scientific studies accessible via the following link: <https://www.insee.fr/fr/information/2008466>

- Santé Publique France data can also be used free of charge for scientific accessible via the following link: studieshttps://www.santepubliquefrance.fr/footer/mentions-legales#block-558639

- IGN map data are available under ODBL license, and can be used free of charge for accessible via the following link: scientific studies <https://www.data.gouv.fr/fr/datasets/carte-des-departements-2-1/>
