# Supplementary material for: Relying on the French territorial offer of thermal spa therapies to build a care pathway for long COVID-19 patients
Source: PLoS One. 2024 Apr 19;19(4):e0302392. doi: 10.1371/journal.pone.0302392 (PMC11029631; doi:10.1371/journal.pone.0302392)
Supplement: S2 Appendix — (DOCX) [file pone.0302392.s002.docx]

**S2 Appendix. Characteristics of the waters of spas specialized in ENT and respiratory, rheumatological and psychosomatic pathologies**

| **City** | **Spa** | **PSY^1^** | **RH^2^** | **ENT^3^** | **Type ofwater** |
| --- | --- | --- | --- | --- | --- |
| Aix-les-Bains | Les Thermes Marlioz | No | Yes | Yes | Hot water: sulphurous, calcium rich in trace elements; Cold water: calcium bicarbonate and diuretic |
| Aix-les-Bains | Les Thermes Chevalley | No | Yes | No | Hot water: sulphurous, calcium rich in trace elements; Cold water: calcium bicarbonate and diuretic |
| Allevard-les-Bains | Etablissement thermal d'Allevard | No | Yes | Yes | Sulphuric, chlorinated, sodium, calcium, carbogaseous and hydrogen sulphide water |
| Amélie-les-Bains | Etablissement thermal d'Amélie-les-Bains | No | Yes | Yes | Hyperthermal, sulphide, sodium, chlorinated, silica-rich water |
| Amnéville-les-Thermes | Centre thermal Saint-Eloy | No | Yes | Yes | Chlorinated waters, strongly mineralized, rich in trace elements |
| Argelès-Gazost | Etablissement thermal d'Argelès-Gazost | No | No | Yes | Sodium chlorinated water |
| Ax-les-Thermes | Eurothermes | No | Yes | Yes | Hyperthermal sulphuric and sodium waters |
| Bagnères-de-Bigorre | Les Grands Thermes | Yes | Yes | Yes | Calcium sulphated water, high in magnesium |
| Bagnoles-de-l'Orne | Etablissement thermal de Bellevue-la-Reine | No | Yes | No | Oligometallic, chlorinated, sodium, sulphated waters rich in trace elements |
| Bagnols-les-Bains | Etablissement thermal de Bagnols-les-Bains | No | Yes | Yes | Sulphide water, sodium bicarbonate |
| Bains-les-Bains | Thermes de Bain-les-Bains | No | Yes | No | Sodium, calcium bicarbonate waters, rich in silica and oligometallic elements |
| Balaruc-les-Bains | Etablissement thermal municipal de Balaruc-les-Bains | No | Yes | No | Sodium, sulphate, calcium, and magnesium chlorinated waters. Rich in bicarbonates and trace elements |
| Barbotant-les-Thermes | Etablissement thermal de Barbotan | No | Yes | No | Oligometallic, calcium, silicate, and magnesium bicarbonate water |
| Barèges-Barzun | Thermes de Barzun | No | Yes | Yes | Sulphide, sodium, very alkaline waters. Rich in silica and barégine |
| Beaucens | Etablissement thermal de Beaucens | No | Yes | No | Sodium, calcium and fluorinated chlorinated water |
| Berthemont-les-Bains | Etablissement thermal de Berthemont | No | Yes | Yes | Sulphide, sodium, warm, silica-rich waters |
| Bourbon-Lancy | Etablissement thermal de Bourbon-Lancy | No | Yes | No | Bicarbonate, mixed, chlorinated and sodium water |
| Bourbon-l'Archambault | Etablussements thermaux de Bourbon-l'Archambault | No | Yes | No | Sodium, bicarbonate, mixed, bromo-iodide chlorinated water. Presence of mineral salts and trace elements |
| Bourbonne-les-Bains | Etablissement thermal de Bourbonne-les-Bains | No | Yes | Yes | Hyperthermal, sodium, sulphate, calcium and oligopolymetal water |
| Brides-les-Bains | Thermes de Brides-les-Bains | No | Yes | No | Sulphated sodium and calcium water |
| Cambo-les-Bains | Thermes de Cambo-les-Bains | No | Yes | Yes | Sulphide, calcium sulphate and magnesium water |
| Capvern-les-Bains | Etablissement thermal de Capvern | No | Yes | No | Calcium sulphated water, rich in magnesium and trace elements |
| Casteljaloux | Etablissement thermal de Casteljaloux | No | Yes | No | Chlorurosulphated, bicarbonated water with iron, magnesium and trace elements |
| Cauterets | Etablissement thermal César | No | Yes | Yes | Sodium chlorurosulphide waters, rich in silica and trace elements |
| Challes-les-Eaux | Chaîne thermale du Soleil | No | No | Yes | The most sulphurized water in Europe. Sodium, strong, bicarbonate, iodide, and bromide water |
| Châteauneuf | Thermes de Châteauneuf-les-Bains | No | Yes | No | Carbogasses, sodium bicarbonates, ferruginous, manganese-containing water with low mineralization |
| Châtel-Guyon | Etablissement thermal de Châtel-Guyon | No | Yes | No | Carbonated, chlorobicarbonated, sodium, calcium, magnesium-rich water |
| Chaudes-Aigues | Eurospa Chaudes-Aigues | No | Yes | No | Radioactive, sodium, hyperthermal and gaseous water |
| Contrexéville | Etablissement thermal de Contrexéville | No | Yes | No | Calcium sulphate water, rich in magnesium and trace elements |
| Cransac-les-thermes | Etablissement thermal privé de Cransac | No | Yes | No | Sulphated, mixed, clacal, sodium and magnesium water |
| Dax | Thermes Bains Saint-Pierre | No | Yes | No | Hyperthermal, calcium sulphate, sodium chloride and strong water |
| Divonne-les-Bains | Etablissement thermal de Divonne-les-Bains | Yes | Yes | No | Calcium bicarbonate waters, rich in trace elements |
| Enghien-les-Bains | Thermes d'Enghien-les-Bains | No | No | Yes | Sulphurous water |
| Eugénie-les-Bains | Thermes d'Eugénie-les-Bains | No | Yes | No | Sulphurous water |
| Evaux-les-Bains | Grand Hôtel thermal d'Evaux-les-Bains | No | Yes | No | Hyperthermal, sodium sulphate, low mineralized and radioactive water |
| Evian-les-Bains | Etablissement thermal | No | Yes | No | Calcium bicarbonate waters, rich in magnesium and trace elements |
| Jonzac | Etablissement thermal de Jonzac | No | Yes | Yes | Sulphurized, sulphated, mixed, sodium chlorinated water, rich in trace elements |
| La Bourboule | Les Grands Thermes | No | No | Yes | Bicarbonate, sodium chlorides water, rich in trace elements |
| La Chaldette | Etabluissement thermal de la Chaldette | No | No | Yes | Sodium, radioactive, bicarbonate water containing magnesium and trace elements |
| La Léchère | Société des Eaux Thermales de La Léchère | No | Yes | No | Hyperthermal, sulphated, calcium and magnesium water |
| La Preste-les-Bains | Etablissement thermal de la Preste | No | Yes | No | Sulphated, sodium, sulphides waters, rich in silica |
| Lamalou-les-Bains | Etablissement thermal de Lamalou-les-Bains | No | Yes | No | Calcium and sodium, hyperthermal, carbogaseous waters containing magnesium, potassium, zinc and silica |
| Le Mont-Dore | Etablissement thermal du Mont-Dore | No | Yes | Yes | Water richest in silica in France. Sodium bicarbonates, with rare gases water |
| Lectoure | Etablissement thermal de Lectoure | No | Yes | No | Sodium chlorinated water |
| Les Camoins-les-Bains | Etablissement thermal des Camoins | No | Yes | Yes | Sulphated calcium, sulphide, cold water, rich in magnesium and trace elements |
| Les Eaux Bonnes | Station thermale des Eaux Bonnes | No | Yes | Yes | Sulphide, mixed, calcium and sodium chloride water, rich in iodine and glairine |
| Les Eaux Chaudes | Station thermale des Eaux Chaudes | No | Yes | Yes | Sodium, calcium and silicate sulphide waters |
| Les Fumades | Etablissement thermal Sogatherm Fumades Bains | No | Yes | Yes | Calcium sulphated water, rich in magnesium, sulphides, bicarbonates and carbogasses |
| Lons-le-Saunier | Etablissement thermal de Lons | No | Yes | No | Sodium, magnesium, heavy or medium chlorinated water |
| Luchon | Etablissement thermal de Bagnères-de-Luchon | No | Yes | Yes | Unspecified |
| Luxeuil-les-Bains | Etablissement thermal de Luxeuil | No | Yes | No | Hyperthermal, sodium, alkaline, sulphate, fluorine, magnesium and oligometallic water |
| Luz-Saint-Sauveur | Thermes de Luz-Saint-Sauveur | No | No | Yes | Sodium, alkaline sulphide water, rich in rare gas and barégine |
| Molitg-les-Bains | Etablissement thermal de Molitg-les-Bains | No | Yes | Yes | Sodium sulphide water, rich in thermal plankton, slightly radioactive |
| Montbrun-les-Bains | Etablissement thermal de Montbrun-les-Bains | No | Yes | Yes | Sulphide water of calcium, magnesium, of fairly high mineralization |
| Montrond-les-Bains | Parc thermal de Montrond-les-Bains | No | Yes | No | Bicarbonate, carbogaseous and sodium water |
| Morsbronn-les-Bains | Etablissement thermal de Mosrbronn | No | Yes | No | Calcium sulphate, sodium chlorinated water |
| Néris-les-Bains | Exploitation du thermalisme et du tourisme de Néris-les-Bains | Yes | Yes | No | Bicarbonate waters, sodium sulphate, rich in trace elements and gas |
| Neyrac-les-Bains | Maison de Cure de Neyrac | No | Yes | No | Calcium and sodium, magnesium and carbogaseous bicarbonate water |
| Niederbronn-les-Bains | Etablissement thermal de Niederbronn | No | Yes | No | Roman source: chlorinated, sodium and carbogaseous water |
| Pietrapola | Etablissement thermal de Pietrapola | No | Yes | No | Sodium, hyperthermal, predominantly carbonated and chlorinated sodium sulphide water |
| Plombières-les-Bains | Société thermale de Plombières | No | Yes | No | Sulphate, bicarbonate, sodium, calcium water, rich in silica and fluorine |
| Préchacq-les-Bains | Etablissement thermal de Préchacq-les-Bains | No | Yes | Yes | Hyperthermal, sulphate, calcium and sulphurous waters, rich in magnesium |
| Rennes-les-Bains | Thermes de la Haute Vallée | No | Yes | No | Sulphated, calcium, chlorinated, mixed, magnesium and ferruginous water |
| Rochefort | Etablissement thermal La Source de l'Empereur | No | Yes | No | Sulphated, ferriginous water, rich in minerals and oligopolumetallic elements |
| Royat | Etablissement thermal de Royat | No | Yes | No | Sodium, chlorinated and carbogaseous bicarbonate water |
| Saint-Amand-les-Eaux | Etablissement thermal de Saint-Amand-les-Eaux | No | Yes | Yes | Calcium, sulphide, magnesium and bicarbonate waters, with high mineralization |
| Saint-Gervais-les-Bains | Thermes de Saint-Gervais-les-Bains | No | No | Yes | Chlorinated, sulphated, sodium and bromide water |
| Saint-Honoré-les-Bains | Etablissement thermal de Saint-Honoré-les-Bains | No | Yes | Yes | Sulphide water and sodium chloride |
| Saint-Lary-Soulan | Thermes de Saint-Lary-Soulan | No | Yes | Yes | Chlorinated waters rich in silica, sulphides, sulphates and oligometallics |
| Saint-Laurent-les-Bains | Thermes de Saint-Laurent-les-Bains | No | Yes | No | Sodium and oligometallic bicarbonate water |
| Saint-Paul-lès-Dax | Thermes de Sourceo | No | Yes | No | Water of constant composition of chlorosulphate, bicarbonate, sodium, and calcium |
| Salies-de-Béarn | Thermes de Salies-de-Béarn | No | Yes | No | Sodium, strong, bromo-iodide, magnesium and ionized chlorinated water |
| Salies-de-Salat | Société des établissements thermaux de Salies-du-Salat | No | Yes | No | Sodium, strong chlorinated water, bromo-iodide, and magnesium |
| Salins-les-Bains | Etablissement thermal de Salins-les-Bains | No | Yes | No | Hydrochloric water, strong, high in trace elements |
| Saubusse-les-Bains | Etablissement thermal de Saubusse | No | Yes | No | Sodium chlorinated water |
| Saujon | Etablissement thermal de Saujon | Yes | No | No | Sodium chlorinated waters, rich in magnesium and trace elements |
| Thonon-les-Bains | Etablissement thermal de Thonon-les-Bains | No | Yes | No | Mixed, bicarbonated, magnesium-rich water |
| Uriage-les-Bains | Etablissement thermal d'Uriage | No | Yes | Yes | Sulphide, sodium chlorides water with molecular concentration similar to human blood serum |
| Vals-les-Bains | Etablissement thermal de Vals-les-Bains | No | Yes | No | Sodium bicarbonate water |
| Vernet-les-Bains | Etablissement thermal de Vernet-les-Bains | No | Yes | Yes | Sulphide water, sodium, silicate, containing thermal plankton |
| Vichy | Grand Etablissement Thermal de Vichy | No | Yes | No | Sodium, carbogaseous bicarbonate water, rich in mineral salts and trace elements |
| Vittel | Etablissement thermal de Vittel | No | Yes | No | Grande source: sulphate and calcium water; Hépar: calcium sulphate water, rich in magnesium |

^1^PSY : psychosomatic pathologies

^2^RH : rheumatological pathologies

^3^ENT : ear-nose-throat and respiratory pathologies

Source: Officiel Thermalisme [<https://www.officiel-thermalisme.com/2020/04/21/leau-dans-tous-ses-etats-leau-thermale/>
